# Imetelstat therapy in refractory anemia with ring sideroblasts with or without thrombocytosis

**DOI:** 10.1038/bcj.2016.13

**Published:** 2016-03-11

**Authors:** A Tefferi, A Al-Kali, K H Begna, M M Patnaik, T L Lasho, A Rizo, Y Wan, C A Hanson

**Affiliations:** 1 Division of Hematology, Mayo Clinic, Rochester, MN, USA; 2 Janssen Research & Development, LLC, Raritan, NJ, USA; 3 Division of Hematopathology, Mayo Clinic, Rochester, MN, USA

The telomerase inhibitor imetelstat (Janssen Biotech Inc., Raritan, NJ, USA) is a 13-mer lipid-conjugated oligonucleotide that targets the RNA template of human telomerase reverse transcriptase. In a pilot study,^1^ imetelstat produced complete clinical and molecular remissions in myelofibrosis patients with *SF3B1* or *U2AF1* mutations; this provided the rationale for the current study (Clinicaltrials.gov NCT01731951), as such spliceosome mutations are closely associated with refractory anemia with ring sideroblasts with (RARS-T) or without (RARS) thrombocytosis.^2^

The current signal-seeking study included nine patients with RARS-T (*n*=5), RARS (*n*=3) or myelodysplastic/myeloproliferative neoplasm with spliceosome mutation (*n*=1). Diagnoses were according to the World Health Organization criteria.^3^ Because of concern regarding myelosuppression,^1^ the 2-h intravenous infusion of imetelstat was administered at a reduced dose level (7.5 mg/kg every 4 weeks instead of 9.4 mg/kg every 3 weeks). Drug activity was monitored by both formal response criteria^4^ and effect on spleen size, thrombocytosis and leukocytosis. Adverse events were monitored by Common Terminology Criteria for Adverse Events (Version 4.03; US National Cancer Institute, Bethesda, MD, USA). Laboratory correlative studies included analysis of mutations.

As of 10 May 2015, accrual to the study was complete (total nine patients) and median time from study registration was 17.1 months (range, 16.1–20.1). Median age of the study population was 70 years (78% males); eight patients were transfusion-dependent and one patient with RARS-T had marked splenomegaly that was palpable at 16 cm below the left costal margin. Three patients each had leukocytosis and thrombocytosis. Seven patients had received prior treatments, including erythropoiesis-stimulating agents in six patients. Karyotype was abnormal in one patient, with loss of Y chromosome.

Four (44%) patients remain on active treatment, and the five treatment discontinuations were due to death during active therapy (unrelated to imetelstat), discovery of second malignancy, progression into acute leukemia and insufficient response (*n*=2). Median treatment duration was 13.7 months (range, 6.6–17.9). Treatment-emergent grade 4 neutropenia and thrombocytopenia were seen in 2 (22%) and 1 (11%) patients, respectively; grade 3 anemia, neutropenia and thrombocytopenia were seen in 6 (67%), 4 (44%) and 2 (22%) patients, respectively. None of the grade ⩾3 hematologic toxicities lasted ⩾4 weeks, and all demonstrated reversibility. The grade 3 non-hematologic events of aspiration, fatigue and lipase increase occurred in one patient, and a patient with preexisting cardiovascular disease experienced a grade 5 heart failure. Treatment-emergent liver function test abnormalities affected the following: alanine aminotransferase (grade 1, 56%/grade 2, 11%); aspartate transaminase (grade 1, 56%/grade 2, 11%); alkaline phosphatase (grade 1, 33%); and total bilirubin (grade 2, 11%). For patients who discontinued treatment with imetelstat, these events were mostly reversible.

Three (38%) of eight transfusion-dependent patients became transfusion-independent at a median time of 11 weeks (range, 9–14) and their response lasted for a median of 28 weeks (range, 9–37; Figure 1); one of the three patients also had resolution of leukocytosis and thrombocytosis. A fourth patient experienced >50% decrease in palpable spleen size (16 cm at baseline) and a decrease in transfusion need. Two additional patients with thrombocytosis and leukocytosis experienced normalization of counts. Patients were screened before and after imetelstat therapy for mutations involving *JAK2*, *SF3B1*, *U2AF1* and *SRSF2*. Three patients were mutated for *JAK2*, seven for *SF3B1* (4 K700E, 2 H662Q and 1 K666N) and one for *U2AF1* (Q157P). All three anemia responders (H662Q, K700E and K666N) and the fourth patient with spleen response (K700E) were *SF3B1* mutated. Post-treatment analysis showed no effect on mutations.

The current study suggests drug activity in imetelstat-treated patients with RARS or RARS-T. The meager depth of response in the current study, compared with the aforementioned report in myelofibrosis,^1^ might reflect either different disease biology or suboptimal drug dose. Further investigation on the therapeutic benefit of imetelstat in RARS/RARS-T is warranted.

## Figures and Tables

**Figure 1:**
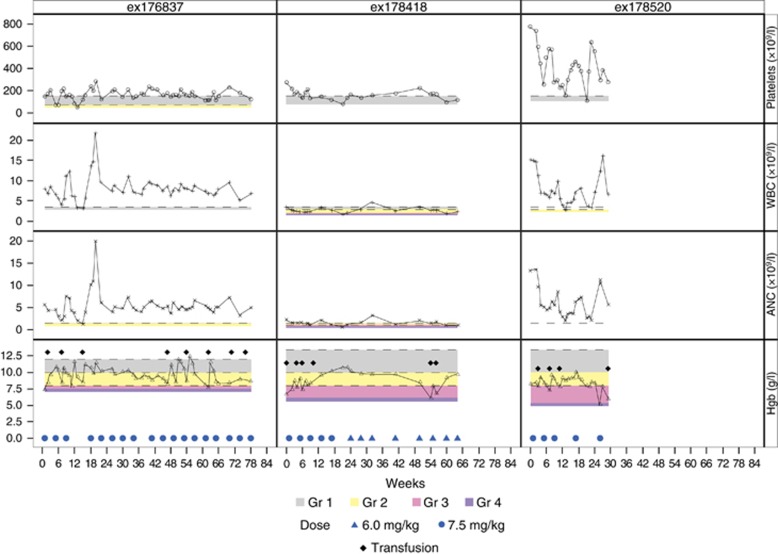
Effect of imetelstat on hemoglobin (Hgb), platelet and leukocyte counts in three patients with refractory anemia with ring sideroblasts with (ex178520) or without (ex176837 and ex178418) thrombocytosis. ex176837: T1 duration=28 weeks and treatment ongoing; ex178418: T1 duration=37 weeks and treatment ongoing; ex178520: T1 duration=9 weeks and off-study July 2014. ANC, absolute neutrophil; WBC, white blood cell.
